# Exploring the mechanism of *Synechococcus* sp. XM-24 in gastric cancer treatment via network pharmacology and molecular docking

**DOI:** 10.1371/journal.pone.0326664

**Published:** 2025-07-02

**Authors:** Jiayi Li, Yamei Ma, Longzhi Guo, Junhong Liu, Lin Li

**Affiliations:** 1 Department of Geriatric Medicine, The Henan Rongjun Hospital, Xinxiang, Henan, China; 2 Department of Electrocardiography and Cardiac Function, The Fuyang Affiliated Hospital of Anhui Medical University, Fuyang, Anhui, China; 3 Department of Gastroenterology, The Fuyang Affiliated Hospital of Anhui Medical University, Fuyang, Anhui, China; 4 Department of Endocrinology, Central Hospital of Haining, Jiaxing, Zhejiang, China; King Abdulaziz University, SAUDI ARABIA

## Abstract

**Objective:**

We investigated the potential molecular mechanisms of *Synechococcus* sp. XM-24 affect gastric cancer (GC) development. Furthermore, this study aimed to provide a theoretical basis for developing novel therapeutic drugs for treating GC.

**Methods:**

The interactions between *Synechococcus* sp. XM-24 and targeted proteins in GC were analyzed through network pharmacology and molecular docking. Molecular dynamics (MD) simulation of the protein–small molecule complex obtained from molecular docking were performed using the Gromacs v2022.03 software. Based on the intersecting target genes of *Synechococcus* sp. XM-24 and GC, Gene Ontology function and Kyoto Encyclopedia of Genes and Genomes analyses were performed to obtain the associated biological processes (BP), cellular components (CC), molecular functions (MF), and signal transduction pathways.

**Results:**

In total, 609 intersecting targets were identified between *Synechococcus* sp. XM-24 and GC, with nine key target genes (*AKT1*, *ALB*, *IL1B*, *SRC*, *STAT3*, *EGFR*, *HSP90AA1*, *ESR1*, and *BCL2*) being identified as active components of *Synechococcus* sp. XM-24. These key target genes were involved in 1,028 BP, 110 CC, 312 MF, and 200 signaling pathways. The enriched signaling pathways mainly included the cancer pathway, metabolic pathway, phosphatidylinositol 3-kinase–protein kinase B(PI3K-AKT) signaling pathway, and Rat sarcoma signaling pathway. Additionally, molecular docking analysis revealed strong binding activities between 10 active components of *Synechococcus* sp. XM-24, including methyl vaccenate, allyl methallyl ether, and 11-octadecenoic acid, with key target proteins such as albumin (ALB). MD simulations demonstrated a stable binding of ALB and methyl vaccenate, with a binding free energy of −48.39 kcal/mol.

**Conclusions:**

Overall, the findings of this study reveal the therapeutic potential of *Synechococcus* sp. XM-24 in the prevention and treatment of GC, along with providing a theoretical basis for further development in GC treatment employing *Synechococcus* sp. XM-24.

## 1. Introduction

Gastric cancer (GC) represents a significant health challenge, with GLOBOCAN 2020 reporting over 1 million new cases and approximately 769,000 deaths. It ranks as the fifth most prevalent cancer diagnosis and the fourth leading cause of cancer-related mortality worldwide [1,2]. Primarily composed of adenocarcinomas (>95%), GC is typically classified based on its anatomical location and histological characteristics [3]. It presents with a poor prognosis owing to late diagnosis at an advanced stage. Obesity, lifestyle, infections with *Helicobacter pylori* and Epstein–Barr Virus are some of the risk factors for GC onset. While the incidence and mortality rates associated with GC have been declining globally owing to increased prevention awareness and advances in screening and treatment strategies, GC still poses a global threat [4].

Treatment modalities, such as surgery, chemotherapy, molecular targeted therapy, or combination approaches, can improve survival and quality of life; however, they exhibit limited efficacy in treating advanced GC. For example, patients have been reported to present local recurrence or distant metastasis (55%–60%) post-resection [5]. Additionally, the resistance of tumor cells to anticancer drugs and the notable side effects of existing drugs necessitate finding alternative therapies that utilize natural compounds and metabolites [6,7]. Approximately, 60% of anticancer drugs presently on the market are isolated from natural sources, particularly marine microbes and plants [8].

The oceans, a vast and underexplored reservoir of marine species, are teeming with natural products possessing potential therapeutic effects [9,10]. These have led to an increase in the scientific interest in exploiting marine natural products and their hidden potential. To date, over 30,000 marine natural products have been discovered, making marine-based drug discovery a global hotspot for drug discovery and development [11]. The discovery of numerous chemically distinct metabolites of different cyanobacterial species with the ability to inhibit the growth and progression of tumor cells by inducing apoptosis has garnered notable attention [12–14]. As a common type of cyanobacteria, *Cyanococcus* spp. exhibit a wide distribution, thriving in colder, nutrient-rich waters to tropical and subtropical regions [15,16]. Previous studies have reported antioxidant and proapoptotic properties in extracts of *Synechococcus* sp. VDW, highlighting them as a valuable source of natural antioxidants and antitumor agents [17]. Similarly, *Synechococcus* 7942 has shown potential as a sensitizer in breast cancer therapy by modulating oxidative stress and apoptosis [18]. Notably, oxidative stress and chronic inflammation play important roles in tumor progression, including gastric, liver, and lung cancers. Active compounds derived from *Polychlorophyllum* sp. can exhibit various biological potentials; however, their therapeutic potential for GC remains unexplored. Similarly, the therapeutic effect of *Synechococcus* sp. XM-24, a member of the Polychlorophyta, on GC, remains unclear. Presently, our understanding of the therapeutic potential and pharmacological mechanisms of marine *Polychlorophyllum* spp. to treat GC is limited.

Network pharmacology is a systematic approach that elucidates drug–disease interactions by constructing a network of “drug components-targets-signalling pathways-therapeutic mechanisms.” This approach aids in understanding the mechanisms of Traditional Chinese Medicine in treating complex diseases and guide drug research and development [19]. Molecular docking and molecular dynamics (MD) simulations are employed to assess receptor–ligand interactions, predicting receptor–ligand binding patterns and affinities [20]. In recent years, network pharmacology and molecular docking techniques have been extensively utilized to identify active compounds and their mechanisms of action. This study aimed to analyze the possible mechanisms of GC inhibition by *Synechococcus* sp. XM-24 utilizing network pharmacology. Additionally, molecular docking and MD simulations were performed to validate these predictions.

## 2. Methods

**Fig 1:**
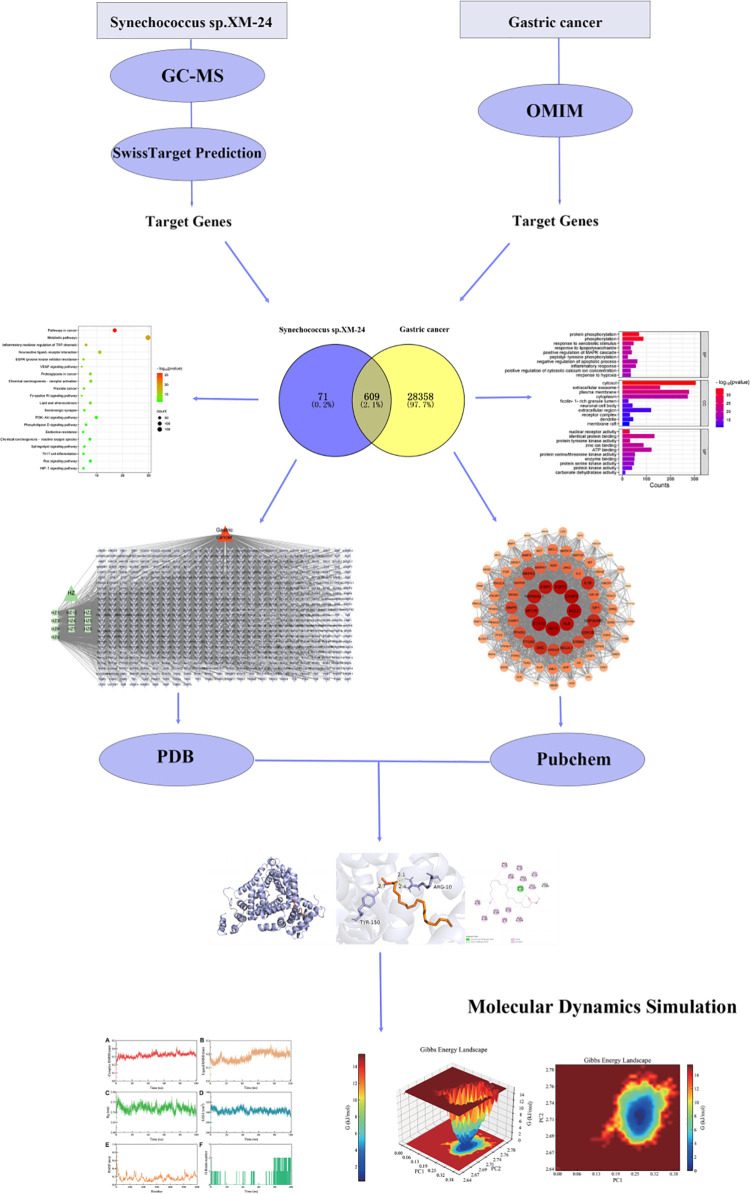
Integrative workflow for study.

The workflow diagram is shown in Fig 1.

### 2.1. *Synechococcus* sp. XM-24 bioactive components and target information acquisition

*Synechococcus* sp. XM-24 biomass was analyzed using pyrolysis–gas chromatography–mass spectrometry [21]. After identifying all the components, the PubChem database was used to exclude components containing toxic substances. Subsequently, Swiss Target Prediction, PharmMapper, and Search Server were used to identify potential targets of the seaweed components. All the data required for this study are from public databases. Ethical approval and consent to participate are not applicable.

### 2.2. GC target acquisition

The GeneCards – Human Genes database, Online Mendelian Inheritance in Man database, and Comparative Toxicogenomics Database were searched using the term “Gastric Cancer” to identify disease-related target genes.

### 2.3. Intersection target acquisition

The identified drug targets and disease targets were analyzed using the Venn Diagramming Software Venn 2.1 to determine the intersecting targets. They were considered as the predicted targets of the drugs acting on the diseases for subsequent analyses.

### 2.4. Protein*–*protein interaction (PPI) network diagram construction and key target screening

The drug–disease intersection targets were fed into the Search Tool for the Retrieval of Interacting Genes/Proteins (STRING) database to construct a PPI network, specifying “*Homo sapiens*” as the organism species. A PPI network with a confidence level of 0.400 was generated. The resulting TSV file from the STRING database was imported into the Cytoscape 3.9.0 software for the subsequent topological analysis.

### 2.5. *Synechococcus* sp. XM-24–GC–target network construction

To gain a comprehensive understanding of the intricate interactions among seaweed, its active components, and their corresponding targets against GC, a network diagram was constructed. This network diagram integrated the seaweed active components and their GC-related intersection targets.

### 2.6. Gene Ontology (GO) enrichment analysis

Biological processes (BP), cellular components (CC), and molecular functions (MF) were determined by subjecting the drug–disease intersection targets to GO enrichment analysis, using DAVID Bioinformatics Resources. The enrichment conditions were as follows: P-value cut-off = 0.05 and Q-value cut-off = 0.05.

### 2.7. Kyoto Encyclopedia of Genes and Genomes (KEGG) pathway enrichment analysis

KEGG pathway enrichment analysis was performed on drug–disease intersection targets using DAVID Bioinformatics Resources, and pathways with P < 0.05 correction were considered significant.

### 2.8. Molecular docking

The Structured Data File of the main active ingredients of the core drugs were retrieved from the PubChem database. Protein structures of key targets were obtained from the Protein Data Bank database and optimized using the PyMOL-2.1.0 software. Water molecules and small ligands were removed, followed by hydrogenation and charge assignment using AutoDockTools-1.5.6. These structures were saved in pdbqt format. Molecular docking was performed using VINA 2.0 within the PYRX software. The key target served as the receptor, and the corresponding active ingredient as the ligand. The docking grid was centered on the ligand coordinates to ensure accurate sampling of the known binding pocket. Binding energies were calculated and output results were generated. Finally, the PyMOL software was used to visualize the results. The affinity (kcal/mol) value represented the binding strength between the ligand and the receptor with lower values indicating stronger and more stable binding. PyMOL was also used to visually analyze its meridians.

### 2.9. MD simulation

The Gromacs v2022.03 software was employed to simulate the 100 ns MD force field of the protein–small molecule complexes obtained from molecular docking using AMBER99SB-ILDN force field [22,23].

Energy minimization was carried out using the Steepest Descent algorithm [24]. The Berendsen constant pressure pump maintained a constant pressure of 1 bar, and the V-rescale thermostat regulated the temperature. The protein structure was pre-balanced at 100 ps optimizing its initial conformation in the solvent. During equilibration in the isothermal and isovolumetric ensemble, the protein–solvent system was heated to the desired temperature for 100 ps. To balance the pressure of the solvent and protein system, 100 ps isothermal isobaric equilibration was done. Finally, root mean square deviation (RMSD), root mean square fluctuation (RMSF), radius of rotation (Rg) values, solution accessible surface area (SASA), and hydrogen bonds (H-bonds) based on the MD simulation trajectory were calculated. According to RMSD and Rg values, Gibbs free energy was calculated using “g_sham” and “xpm2txt.py” scripts built in the Gromacs v2022.03 software. Additionally, the “MMPBSA.py v.16.0” script was used to calculate molecular mechanics/Poisson–Boltzmann surface area (MM/PBSA) [25]. The MM/PBSA algorithm divides the binding free energy into molecular mechanical free energy (ΔG_gas_) and solvation free energy (ΔG_solvation_).

## 3. Results

### 3.1. *Synechococcus* sp. XM-24, its active ingredients, and its intersecting targets with GC

**Fig 2:**
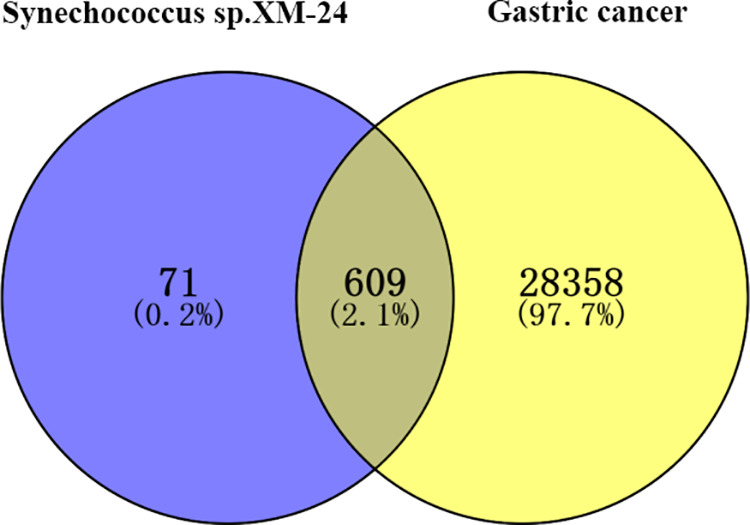
Venn Diagram: Seaweed-Gastric cancer intersection targets.

GC-2012MS analysis identified 42 seaweed components, of which 32 components exhibited potential toxicity. The other 10 seaweed components were selected for further study (Table 1). Additionally, the Swiss Target Prediction, PharmMapper, and SEA database identified 680 targets for the components. Fig 2 illustrates 609 intersecting targets identified for cyanobacteria and GC in this study.

**Table 1:** Compound protein identification.

Number	Compound label	MOL name
1	HZ9	11-Octadecenoic acid, methyl ester
2	HZ10	n-Capric acid isopropyl ester
3	HZ7	3-Undecene, (E)
4	HZ5	3-Undecene, (Z)
5	HZ8	Undecane, 2-methyl
6	HZ2	Allyl methallyl ether
7	HZ1	2H-Pyran-2,4(3H)-dione, dihydro-6-methyl
8	HZ4	1-Heptene, 2-methyl-
9	HZ6	Phthalazine, 1-methyl
10	HZ3	Benzenemethanol, 4-methyl-

### 3.2. Construction of PPI networks and screening of key targets

**Fig 3:**
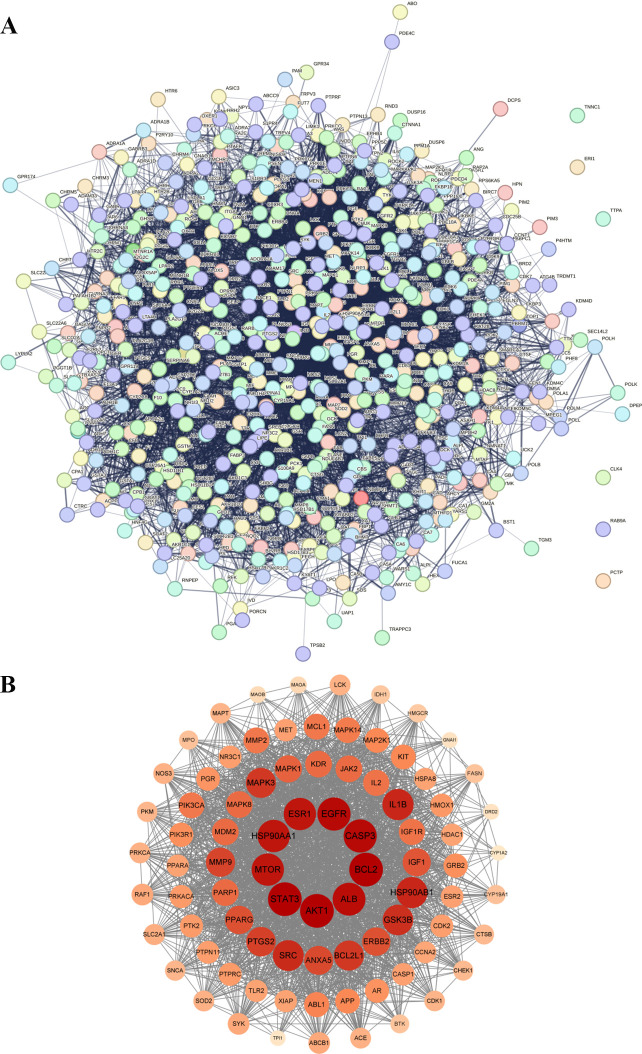
PPI analysis. A: PPI analysis of Seaweed-Gastric cancer intersection targets; B: degree>60 key target protein PPI analysis.

A PPI network was constructed using Cytoscape comprising 601 nodes, 9752 edges, and an average degree of 32.1(Fig 3A). Key target genes (namely *AKT1*, *ALB*, *IL1B*, *SRC*, *STAT3*, *EGFR*, *HSP90AA1*, *ESR1*, and *BCL2*) of the compounds against the diseases were screened according to the degree values (Table 2). Targets with a degree value exceeding 60 were selected to construct the PPI network. High degree values had larger sizes and darker indicators, and lower degree values had smaller sizes and lighter indicators (Fig 3B).

**Table 2:** Screening of key targets.

Number	Protein label	Degree value
1	AKT1	Degree: 227.0
2	ALB	Degree: 213.0
3	IL1B	Degree: 192.0
4	SRC	Degree: 188.0
5	STAT3	Degree: 178.0
6	EGFR	Degree: 177.0
7	HSP90AA1	Degree: 169.0
8	ESR1	Degree: 160.0
9	BCL2	Degree: 158.0

### 3.3. Construction of the seaweed–GC–target network

**Fig 4:**
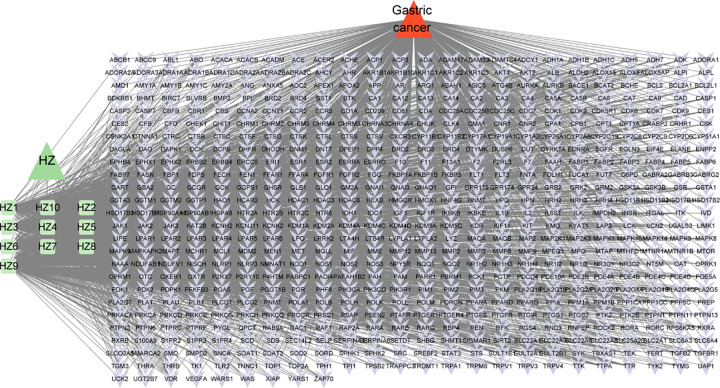
Seaweed-gastric cancer-target network diagram. The green triangle is the seaweed, the green square is the seaweed active ingredient, the red triangle is gastric cancer, and the purple arrow is the intersection target.

To comprehensively understand the intricate relationships between seaweed, its active ingredients, its targets, and GC, a seaweed–GC–target network diagram was constructed. This network included seaweed components, its active ingredients, and the intersecting targets. The Cytoscape 3.9.0 software was used to visualize the network diagram (Fig 4). Based on topological analysis, the top 10 compounds were identified as key compounds (Table 1).

### 3.4. GO analysis

In total, 1,450 GO entries were enriched, including 1,028 BP, 110 CC, and 312 MF. The enriched BP mainly included protein phosphorylation, phosphorylation, response to exogenous stimuli, response to lipopolysaccharide, positive regulation of the mitogen-activated protein kinase (MAPK) cascade, peptide tyrosine phosphorylation, negative regulation of apoptosis, inflammatory response, positive regulation of cytoplasmic calcium ion concentration, and hypoxia reaction among other processes.

**Fig 5:**
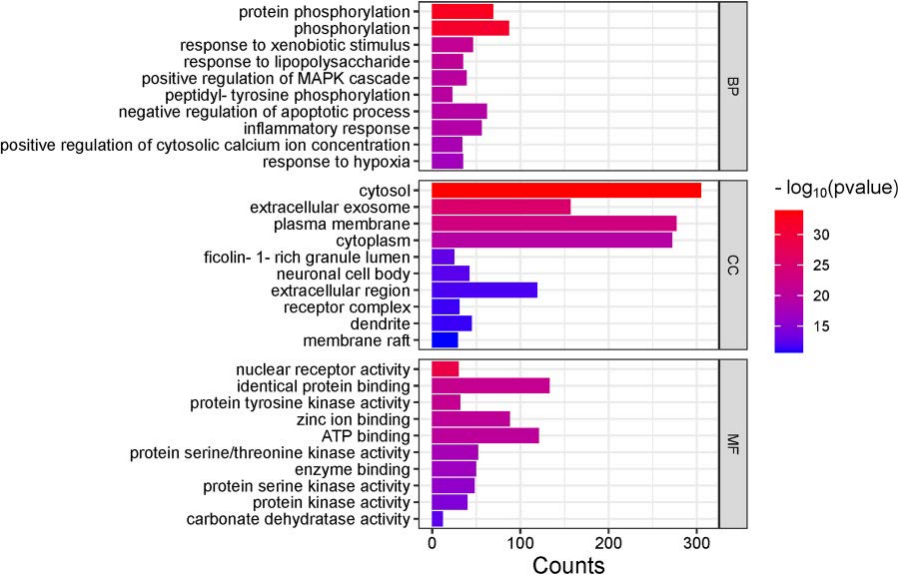
GO analysis: BP, CC, MF. The abscissa represents the number of target genes, the left represents BP, CC, MF, and the color represents the P value, the smaller the P value, the more blue the color is, and the larger the P value, the more red it is.

CC was mainly identified in the cytoplasm, exosomes, plasma membrane, cytoplasm, ficolin-1-rich granular lumen, neuronal cell body, extracellular region, receptor complex, dendrite, and membrane raft among other components. The enriched MF mainly included nuclear receptor activity, identical protein binding, protein tyrosine kinase activity, zinc ion binding, adenosine triphosphate (ATP) binding, protein serine/threonine kinase activity, enzyme binding, protein serine kinase activity, protein kinase activity, and carbonate dehydrase activity among other functions. The top 10 enrichment terms with the highest P-values were selected and visualized using microbiomes according to the P-value of each item and the number of genes enriched within it (Fig 5).

### 3.5. KEGG enrichment analysis

**Fig 6:**
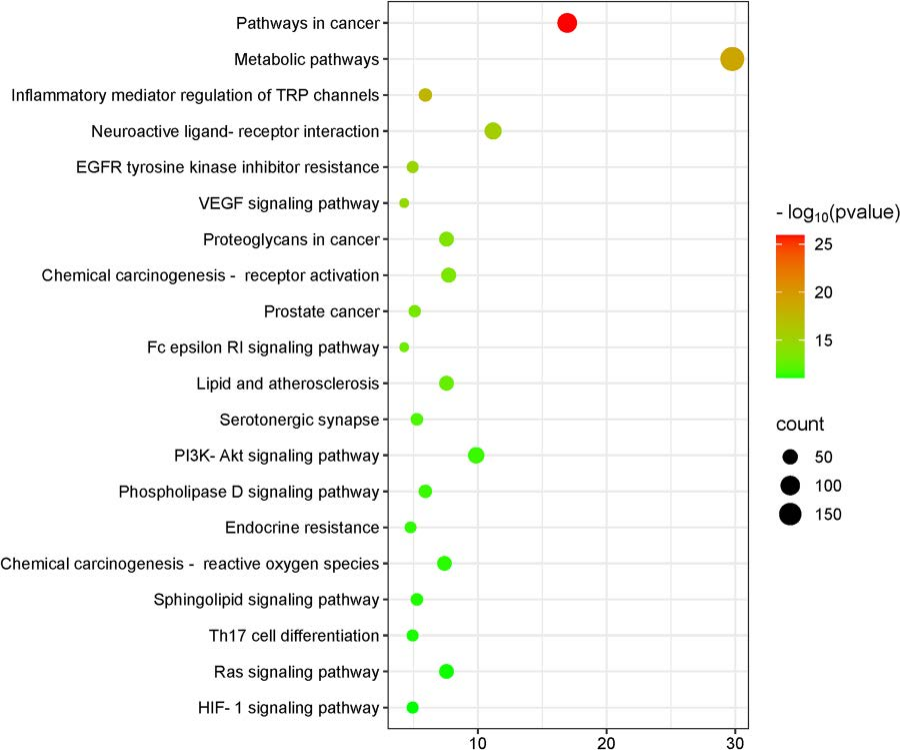
KEGG pathway analysis. The abscissa represents the number of genes enriched, the left side represents the pathway name, and the color represents the P-value, the smaller the P-value, the greener the color, and the larger the P-value, the more red.

KEGG pathway enrichment analysis identified 200 signaling pathways. A microbiome plot was constructed for the top 20 enriched pathways (Fig 6), ranked by P-values and the number of enriched genes.

Notably, the drug–disease intersection targets were significantly enriched in pathways related to cancer, metabolic pathway, inflammatory mediator regulation of TRP channel, neuroactive ligand–receptor interaction, the resistance of epidermal growth factor receptor (*EGFR*) tyrosine kinase inhibitors, vascular endothelial growth factor signaling pathway, proteoglycan in cancer, chemocarno-receptor activation, prostate cancer, FcεRI signaling pathway, lipid and atherosclerosis, serotonergic synapse, phosphatidylinositol 3-kinase (*PI3K*)–protein kinase B (*Akt*) signaling pathway, phospholipase D signaling pathway, endocrine resistance, chemical carcinogenic-reactive oxygen species, sphingolipid signaling pathway, Th17 cell differentiation, Rat sarcoma (Ras) signaling pathway, and hypoxia-inducible factor-1 (HIF-1) signaling pathway among others.

### 3.6. Molecular docking

The PyMOL software was used to visualize the molecular docking results. The binding affinity (kcal/mol) represented the strength of the ligand–receptor interaction, with lower values signifying a more stable binding. Table 2 lists the identified compound proteins, Table 3 lists the identified target proteins, and Table 4 lists the docking affinity results.

**Table 3:** Target protein identification.

Number	Protein label	PDB ID
1	AKT1	1unq
2	ALB	6yg9
3	IL1B	5r8q
4	SRC	1a07
5	STAT3	6njs
6	EGFR	5ug9
7	HSP90AA1	1byq
8	ESR1	7qvl
9	BCL2	6gl8

**Table 4:** Affinity and energy docking results.

Ligand/Protein	AKT1	ALB	IL1B	SRC	STAT3
Methyl vaccenate	−4.2	−6.7	−4.8	−4.8	−4.3
Isopropyl decanoate	−4.1	−6.4	−4.8	−5.0	−4.6
3-Undecene, (E)	−4.4	−6.2	−4.5	−4.3	−4.4
3-Undecene, (Z)	−3.9	−6.4	−4.2	−4.5	−4.1
2-Methylundecane	−3.8	−6.2	−4.6	−3.9	−3.5

**Fig 7:**
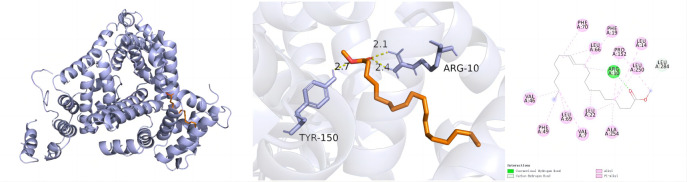
ALB_Methyl vaccenate_complex.

Fig 7 illustrates, molecular docking interactions between albumin (ALB) and methyl vaccenate. ALB interacts with ARG-10, the target protein of methyl vaccenate, to form hydrogen bonds with VAL-46, Ph-49, LEU-69, VAL-7, LEU-22, ALA-254, LEU-250, PRO-152, LEU-14, LEU-66, Ph-19, and Ph-70 forming alkyl and pi-alkyl hydrophobic interactions. The docking analysis revealed a bonding energy of −6.7 kcal/mol between ALB and methyl vaccenate.

### 3.7. RMSD, RMSF, Rg, SASA, and hydrogen bonds in MD simulations

**Fig 8:**
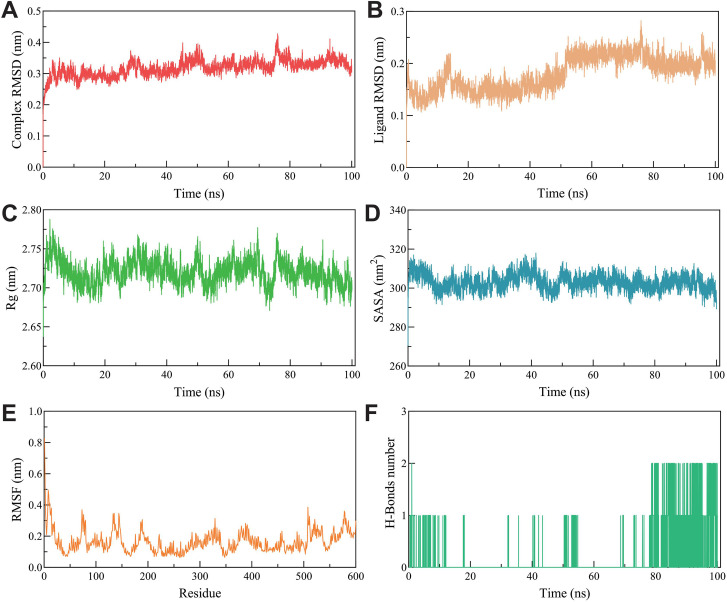
Molecular dynamics simulation analysis of ALB-Methyl vaccenate. **(A)** RMSD curve of ALB, **(B)** RMSD curve of Methyl vaccenate, **(C)** Rg curve of complex, **(D)** SASA curve of complex, **(D)** RMSF curve of protein, and (F) hydrogen bond change curve of complex.

The RMSD curve represents the fluctuation of protein conformation. Fig 8A shows that the RMSD of ALB exhibits fluctuations within a certain range between 0–20 ns. As shown in Fig 8B, the RMSD of methyl vaccenate remained low (<0.3 nm), indicating that it exhibits minimal motion within the binding site and maintains a stable bond.

Fig 8C shows that the Rg value remained stable throughout the simulation (between 2.65 and 2.8 nm), suggesting that the complex structure did not undergo significant changes. Fig 8D shows that SASA exhibited minimal fluctuation, indicating that the protein’s surface solvent accessibility was stable, with no significant opening or closing of the binding pocket. Fig 8E shows that the RMSF values of most residues were low (<0.4 nm), indicating limited flexibility of the protein backbone, which further supported the stability of the complex. Fig 8F shows the fluctuations in the number of hydrogen bonds during the simulation, which stabilized in the range of approximately 1–2 hydrogen bonds. Overall, the stable RMSD values of the complex and ligand indicated that the structure of the system remained unchanged throughout the 100 ns simulation.

### 3.8. Gibbs free energy in MD simulation

**Fig 9:**
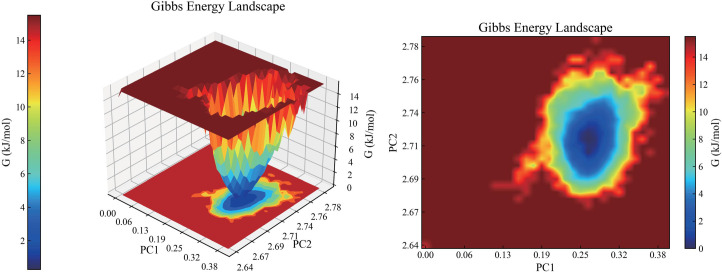
Gibbs free energy of ALB-Methyl vaccenate.

The Gibbs free energy map provides insights into the conformational behavior of protein–ligand interactions. Herein, the blue region on the map represents the lowest free energy, indicating the most stable system (Fig 9). Conversely, the red areas represent higher free energies, indicating less stable systems.

### 3.9. Free energy of complex binding in MD simulations

The interaction between ALB and methyl vaccenate was driven by van der Waals forces and non-polar solvation energy (Table 5), suggesting strong hydrophobicity and shape complementarity. The calculated binding free energy of −48.39 kcal/mol indicated a thermodynamically stable complex with high binding affinity. However, the polar solvation energy (ΔEGB) contributed negatively to the binding free energy, suggesting that the binding site may be dependent on non-polar interactions.

**Table 5:** The Average Binding free Energy of ALB-Methyl vaccenate (kcal/mol).

Energy contributions	ALB-Methyl vaccenate
ΔVDWAALS	−56.27
ΔE_elec_	−2.65
ΔE_GB_	18.82
ΔE_surf_	−8.3
ΔG_gas_	−58.92
ΔG_solvation_	10.53
ΔTotal	−48.39

ΔGgas is divided into van der Waals interaction energy (ΔVDWAALS) and electrostatic energy (ΔEelec). ΔGsolvation is divided into polar solvation energy (ΔEGB) and non-polar solvation energy (ΔEsurf). The total binding free energy (ΔTotal) is the sum of ΔGgas and ΔGsolvation and is an important index reflecting the affinity of the interaction.

## 4. Discussion

GC is a highly lethal disease with a growing incidence and mortality rate worldwide. It exhibits higher morbidity and mortality rates in males (15.8 new cases and 11 deaths/100,000 population/year) compared with those in females (7.0 new cases and 4.9 deaths/100,000 population/year) [26]. In addition to radical surgery, the preferred treatment for GC, many other approaches such as perioperative chemotherapy, adjuvant chemotherapy, adjuvant chemoradiotherapy, targeted therapy, and immune checkpoint inhibitors have been reported to reduce the risk of GC recurrence and improve long-term survival [3,27–29]. Nevertheless, the search for novel therapeutic strategies remains crucial..

By the end of 2022, 17 marine-derived drugs had been approved for clinical use, with 12 of them being approved for cancer treatment highlighting their therapeutic potential and antitumor efficacy [30]. Studies have demonstrated that algae and their extracts can regulate oxidative stress, tumors, and inflammation, with their extracts or derivatives playing an antitumor role, particularly in breast [31], colon [32], and oral cancers [33]. Reportedly, *Synechococcus* can exhibit antitumor effects in breast cancer by inducing cell death or inhibiting growth in sensitizer-mediated photoacoustic kinetic therapy [18]. However, the anti-GC effects of *Synechococcus* derivatives remain unexplored. This study aimed to elucidate the mechanisms of seaweed in treating GC employing network pharmacology and molecular docking.

Network pharmacology, an approach proposed by Hopkins in 2008, enables the prediction of drug targets and their mechanisms of action [34]. In contrast, molecular docking simulates the interactions between drug molecules and protein targets at the atomic level, providing insights into their binding mechanisms [35].

In recent years, network pharmacology and molecular docking have emerged as powerful tools for elucidating the molecular mechanisms underlying drug treatment for cancer. Using this integrated approach, this study aimed to investigate the therapeutic potential of *Synechococcus* sp. XM-24 in GC, contributing to the ongoing discourse on health and disease management.

This study identified 42 active ingredients within *Synechococcus* sp. XM-24 and 609 intersecting targets associated with GC. Of these, the top 10 *Synechococcus* sp. XM-24 active ingredients were screened (Table 1), and nine key target genes (*AKT1*, *ALB*, *IL1B*, *SRC*, *STAT3*, *EGFR*, *HSP90AA1*, *ESR1*, and *BCL2*) were prioritized based on their degree ranking.

Notably, *AKT1* encodes a serine/threonine protein kinase. It is activated by extracellular signaling via a PI3K-dependent mechanism. The AKT signaling pathway regulates cell proliferation, growth, apoptosis, and glucose metabolism, and the aberrant AKT activation leads to tumorigenesis [36–38]. In 2019, Zhang et al. found that circular RNA nuclear receptor interacting protein 1 acts as a sponge for miR-149-5p, leading to increased AKT serine/threonine kinase 1 activity, and ultimately promoting tumor growth in GC [39].

ALB is a highly abundant protein in the blood with a long circulation time. It serves as a natural carrier for ligands and hydrophobic molecules. Rapidly growing and nutrient-deficient cancer cells readily absorb and metabolize ALB [40]. Previous studies, employing network pharmacology and molecular docking, have established a link between cordycepin and its target gene *ALB*. Furthermore, in vitro experiments have verified these findings, demonstrating that cordycepin enhances *ALB* expression in vitro and exhibits anti-breast tumor effects [41]. Reportedly, an increased serum lactate dehydrogenase–ALB ratio is a reliable indicator of poor prognosis in patients with GC treated with nivolizumab [42]. Similarly, the C-reactive protein–ALB ratio is strongly associated with poor prognosis in patients with adenocarcinoma of the esophagogastric junction and upper GC [43].

Interleukin-1B (IL-1B) is a pro-inflammatory cytokine and plays a crucial role in initiating and coordinating inflammatory responses. It influences innate and adaptive immunity, and it can exhibit both antitumor and protumor effects [44].

SRC tyrosine kinase primarily exerts its pro-cancer function by catalyzing the tyrosine phosphorylation of various protein substrates. This activity can promote tumor proliferation, reorganize the actin cytoskeleton of cancer cells, trigger invasion and metastasis, and induce angiogenesis. Signal transducer and activator of transcription 3 (STAT3) is a crucial intracellular signaling molecule involved in various physiological and pathological processes of cells [45].

The interplay between STAT3 and autophagy can have either synergistic or antagonistic roles at different stages of tumor development. This can either promote or inhibit tumor initiation and progression [46,47]. In 2021, Li Zengliang demonstrated that microRNA-653-5p can drive the proliferation and metastasis of GC by targeting the suppressor of cytokine signaling 6–STAT3 pathway [48].

EGFR is a receptor encoded by the proto-oncogene c-erB-1. Furthermore, EGFR mutations can lead to its constant activation, causing uncontrolled cell growth, survival and resistance to apoptosis, leading to tumor formation.

Studies have demonstrated a strong association between the expression of *HSP90AA1* and the malignant phenotype of GC. In primary GC, *HSP90AA1* exhibits increased expression at both the transcriptional and translational levels compared to normal gastric mucosa [49,50]. Research has shown that cancer-associated, fibroblast-derived exosomes promote the viability, migration, and invasion of GC cells by upregulating the interleukin 32/estrogen receptor 1 (ESR1) axis [51].

ESR1 is a key gene encoding estrogen receptor α (ERα). Transcriptional regulation of ESR1 is crucial for mediating the downstream effects of estrogen signaling pathways, particularly cell growth in breast cancer. ERα is the primary target for endocrine therapy because of its critical role in the proliferation and survival of ERα-positive breast cancer [52].

BCL*2*, a key target gene, belongs to the anti-apoptotic B-cell lymphoma 2 (BCL-2) protein family, known to play a crucial role in the development and progression of GC. BCL2 has been reported to be a potential target for therapeutic drugs against GC [53].

Altogether, the key targets identified in this study are primarily involved in tumor apoptosis, proliferation, invasion, and migration. These findings collectively suggest the potential therapeutic efficacy of *Synechococcus* sp. XM-24 and its active ingredients in treating GC at the molecular level.

GO enrichment analysis on the key target genes, revealed 1,450 entries, encompassing 1,028 BP, 110 CC, and 312 MF. Key BP included protein phosphorylation, negative regulation of apoptosis, and inflammatory responses. CC was mainly localized in the cytoplasm, cellular exosomes, ficolin-1-rich granular lumen, and membrane rafts. MF mainly encompassed protein tyrosine kinase activity, ATP binding, protein serine/threonine kinase activity, and enzyme binding.

Apoptosis is a cell death program, and the activation of the apoptotic pathway and their associated molecular targets represent a significant anticancer strategy [54,55]. Reportedly, phosphorylation of enzymes by tyrosine kinases, such as pyruvate dehydrogenase kinase 1, can disrupt mitochondrial pyruvate metabolism in cancer cells [56].

KEGG pathway enrichment analysis identified 200 signaling pathways, with key targets significantly enriched in pathways related to pathogenesis, metabolism, PI3K–Akt signaling, Ras signaling, HIF-1 signaling, and inflammatory mediator regulation of TRP channels.

Frequent activation of the PI3K–Akt pathway has been strongly associated with the occurrence and progression of GC. Inhibition of this pathway is considered a promising therapeutic strategy for GC treatment [57,58].

Mutations in the Ras proto-oncogene are closely linked to various cancers, including bowel and lung cancers. Studies have shown that targeting Cytosolic phospholipase A2 (cPLA2α) inhibits GC and enhances chemotherapy efficacy by suppressing the Ras/MAPK kinase/extracellular signal-regulated kinase and Akt/β-catenin pathways [59].

HIF-1, a nuclear protein encoded by *HIF-1* was first discovered by Semenza and Wang in 1992. This protein binds to target genes and plays a critical role in inflammation and tumor growth. Kotaro Ito et al. showed that matrix metalloproteinase-1 expression is regulated by HIF-1-dependent and epigenetic mechanisms, exerting a tumor-suppressive effect in GC progression [60].

Molecular docking analysis was performed to investigate the interactions between key targets and the active components of *Synechococcus* sp. XM-24. The results showed that most active components exhibited strong binding affinities to their respective targets, with binding energies generally below −4.0 kcal/mol. Notably, ALB and methyl vaccenate presented a favorable binding energy of −6.7 kcal/mol, indicating strong and stable interactions. Discovery Studio 2020 Client was employed for visual analysis of ALB and methyl vaccenate. MD simulation further supported these findings, revealing stable binding conformations for ALB and methyl vaccenate with a total binding free energy of −48.39 kcal/mol. This study provides preliminary evidence for the accuracy of the predicted targets through molecular docking. The results suggest that *Synechococcus* sp. XM-24 may exert anti-cancer effects on GC by modulating these key targets.

## 5. Limitations

While this study provides evidence for the potential anti-GC effects of *Synechococcus* sp. XM-24, it is crucial to acknowledge certain limitations. First of all, these several targeted proteins are involved in basic physiological activities of the human body, such as substance transport and immune regulation. Long-term inhibition of them may have adverse effects on physiological activities in the body. ALB possesses some important physiological functions necessary for normal health, including binding and transporting, maintaining plasma osmotic pressure and scavenging free radicals, accounting for the majority of plasma antioxidant capacity [61]. The activation and release of IL-1B play a key role in initiating and coordinating inflammatory responses. If ALB and IL-1B are inhibited for a long time, it may lead to dysregulation of substance transport and immunity in the body. Variations in the STAT3 gene are associated with increased susceptibility to autoimmune diseases such as psoriasis and multiple sclerosis [62,63]. AKT1 regulates cell proliferation, growth, apoptosis and glucose metabolism. Therefore, the up-regulation and down-regulation of these protein genes can cause a wide range of side effects. EGFR has multiple closely related mutant subtypes. While it is used as a targeted drug for anti-tumor treatment, it often causes skin toxicity [64], enterotoxicity and liver damage. Meanwhile, SRC has multiple kinase family members, and the inhibition of HSP90AA1 triggers a protective heat shock response. This also indicates that these targeted proteins have redundancy when exerting anti-tumor effects, and tumor cells bypass the inhibition through other alternative pathways. The upregulation of BCL2 inhibitors through MCL1 can lead to drug resistance, while STAT3 can reactivate cytokine signals, enabling the disease to evolve mechanisms that evade inhibition. The identification of relevant targets relied on database information, which may be subject to limitations in data accuracy. Further, *in vitro* and *in vivo* experimental validation is essential to fully characterize the therapeutic potential of *Synechococcus* sp XM-24 for its clinical applications.

## 6. Conclusion

Overall, the result suggest that *Synechococcus* sp. XM-24 may exhibit anticancer effects on GC by modulating key cell processes, such as cell cycle regulation, proliferation, invasion, and migration, along with apoptosis. These findings highlight the potential of *Synechococcus* sp. XM-24 in preventing and treating GC, along with a foundation for further in-depth research into its therapeutic applications for GC.

## Supporting information

S1 Table The active ingredient of; *Synechococcus* XM-24. (XLSX)

## References

[1] Sung H, Ferlay J, Siegel RL, Laversanne M, Soerjomataram I, Jemal A, et al. Global Cancer Statistics 2020: GLOBOCAN Estimates of Incidence and Mortality Worldwide for 36 Cancers in 185 Countries. CA Cancer J Clin. 2021;71(3):209–49. doi: 10.3322/caac.21660 33538338

[2] Siegel RL, Miller KD, Fuchs HE, Jemal A. Cancer Statistics, 2021. CA Cancer J Clin. 2021;71(1):7–33. doi: 10.3322/caac.21654 33433946

[3] Ajani JA, D’Amico TA, Bentrem DJ, Chao J, Cooke D, Corvera C, et al. Gastric Cancer, Version 2.2022, NCCN Clinical Practice Guidelines in Oncology. J Natl Compr Canc Netw. 2022;20(2):167–92. doi: 10.6004/jnccn.2022.0008 35130500

[4] Yang W-J, Zhao H-P, Yu Y, Wang J-H, Guo L, Liu J-Y, et al. Updates on global epidemiology, risk and prognostic factors of gastric cancer. World J Gastroenterol. 2023;29(16):2452–68. doi: 10.3748/wjg.v29.i16.2452 37179585 PMC10167900

[5] Ruiz Hispán E, Pedregal M, Cristobal I, García-Foncillas J, Caramés C. Immunotherapy for Peritoneal Metastases from Gastric Cancer: Rationale, Current Practice and Ongoing Trials. J Clin Med. 2021;10(20):4649. doi: 10.3390/jcm10204649 34682772 PMC8539177

[6] Khan SU, Fatima K, Aisha S, Malik F. Unveiling the mechanisms and challenges of cancer drug resistance. Cell Commun Signal. 2024;22(1):109. doi: 10.1186/s12964-023-01302-1 38347575 PMC10860306

[7] Barachini S, Ghelardoni S, Varga ZV, Mehanna RA, Montt-Guevara MM, Ferdinandy P, et al. Antineoplastic drugs inducing cardiac and vascular toxicity - An update. Vascul Pharmacol. 2023;153:107223. doi: 10.1016/j.vph.2023.107223 37678516

[8] Newman DJ, Cragg GM. Natural Products as Sources of New Drugs over the Nearly Four Decades from 01/1981 to 09/2019. J Nat Prod. 2020;83(3):770–803. doi: 10.1021/acs.jnatprod.9b01285 32162523

[9] Carroll AR, Copp BR, Grkovic T, Keyzers RA, Prinsep MR. Marine natural products. Nat Prod Rep. 2024;41(2):162–207. doi: 10.1039/d3np00061c 38285012

[10] Silva M, Avni D, Varela J, Barreira L. The Ocean’s Pharmacy: Health Discoveries in Marine Algae. Molecules. 2024;29(8):1900. doi: 10.3390/molecules29081900 38675719 PMC11055030

[11] Lyu C, Chen T, Qiang B, Liu N, Wang H, Zhang L, et al. CMNPD: a comprehensive marine natural products database towards facilitating drug discovery from the ocean. Nucleic Acids Res. 2021;49(D1):D509–15. doi: 10.1093/nar/gkaa763 32986829 PMC7779072

[12] Shishido TK, Popin RV, Jokela J, Wahlsten M, Fiore MF, Fewer DP, et al. Dereplication of Natural Products with Antimicrobial and Anticancer Activity from Brazilian Cyanobacteria. Toxins (Basel). 2019;12(1):12. doi: 10.3390/toxins12010012 31878347 PMC7020483

[13] Prabakaran G, Sampathkumar P, Kavisri M, Moovendhan M. Extraction and characterization of phycocyanin from Spirulina platensis and evaluation of its anticancer, antidiabetic and antiinflammatory effect. Int J Biol Macromol. 2020;153:256–63. doi: 10.1016/j.ijbiomac.2020.03.009 32142842

[14] Bouyahya A, Bakrim S, Chamkhi I, Taha D, El Omari N, El Mneyiy N, et al. Bioactive substances of cyanobacteria and microalgae: Sources, metabolism, and anticancer mechanism insights. Biomed Pharmacother. 2024;170:115989. doi: 10.1016/j.biopha.2023.115989 38103309

[15] Zwirglmaier K, Jardillier L, Ostrowski M, Mazard S, Garczarek L, Vaulot D, et al. Global phylogeography of marine *Synechococcus* and Prochlorococcus reveals a distinct partitioning of lineages among oceanic biomes. Environ Microbiol. 2008;10(1):147–61. doi: 10.1111/j.1462-2920.2007.01440.x 17900271

[16] Post AF, Penno S, Zandbank K, Paytan A, Huse SM, Welch DM. Long term seasonal dynamics of *synechococcus* population structure in the gulf of aqaba, northern red sea. Front Microbiol. 2011;2:131. doi: 10.3389/fmicb.2011.00131 21734910 PMC3122069

[17] Suttisuwan R, Phunpruch S, Saisavoey T, Sangtanoo P, Thongchul N, Karnchanatat A. Free Radical Scavenging Properties and Induction of Apoptotic Effects of Fa Fraction Obtained after Proteolysis of Bioactive Peptides from Microalgae *Synechococcus* sp. VDW. Food Technol Biotechnol. 2019;57(3):358–68. doi: 10.17113/ftb.57.03.19.6028 31866749 PMC6902293

[18] Zhao R, Zhao P, Zhou Z, Liu D, Zhou Y, Zheng M, et al. Evaluation of the value of *Synechococcus* 7942 as a sensitizer for photo-sonodynamic therapy against breast cancer. Biosci Trends. 2024;18(4):335–42. doi: 10.5582/bst.2024.01211 39168611

[19] Li X, Liu Z, Liao J, Chen Q, Lu X, Fan X. Network pharmacology approaches for research of Traditional Chinese Medicines. Chin J Nat Med. 2023;21(5):323–32. doi: 10.1016/S1875-5364(23)60429-7 37245871

[20] Meng X-Y, Zhang H-X, Mezei M, Cui M. Molecular docking: a powerful approach for structure-based drug discovery. Curr Comput Aided Drug Des. 2011;7(2):146–57. doi: 10.2174/157340911795677602 21534921 PMC3151162

[21] Xie R, Chen F, Ma Y, Hu W, Zheng Q, Cao J, et al. Network pharmacology‒based analysis of marine cyanobacteria derived bioactive compounds for application to Alzheimer’s disease. Front Pharmacol. 2023;14:1249632. doi: 10.3389/fphar.2023.1249632 37927608 PMC10620974

[22] Van Der Spoel D, Lindahl E, Hess B, Groenhof G, Mark AE, Berendsen HJC. GROMACS: fast, flexible, and free. J Comput Chem. 2005;26(16):1701–18. doi: 10.1002/jcc.20291 16211538

[23] Lindorff-Larsen K, Piana S, Palmo K, Maragakis P, Klepeis JL, Dror RO, et al. Improved side-chain torsion potentials for the Amber ff99SB protein force field. Proteins. 2010;78(8):1950–8. doi: 10.1002/prot.22711 20408171 PMC2970904

[24] Donnelly SM, Lopez NA, Dodin IY. Steepest-descent algorithm for simulating plasma-wave caustics via metaplectic geometrical optics. Phys Rev E. 2021;104(2–2):025304. doi: 10.1103/PhysRevE.104.025304 34525672

[25] Genheden S, Ryde U. The MM/PBSA and MM/GBSA methods to estimate ligand-binding affinities. Expert Opin Drug Discov. 2015;10(5):449–61. doi: 10.1517/17460441.2015.1032936 25835573 PMC4487606

[26] Ferlay J, Colombet M, Soerjomataram I, Parkin DM, Piñeros M, Znaor A, et al. Cancer statistics for the year 2020: An overview. Int J Cancer. 2021;:10.1002/ijc.33588. doi: 10.1002/ijc.33588 33818764

[27] Guan W-L, He Y, Xu R-H. Gastric cancer treatment: recent progress and future perspectives. J Hematol Oncol. 2023;16(1):57. doi: 10.1186/s13045-023-01451-3 37245017 PMC10225110

[28] Lordick F, Carneiro F, Cascinu S, Fleitas T, Haustermans K, Piessen G, et al. Gastric cancer: ESMO Clinical Practice Guideline for diagnosis, treatment and follow-up. Ann Oncol. 2022;33(10):1005–20. doi: 10.1016/j.annonc.2022.07.004 35914639

[29] Wang F-H, Zhang X-T, Li Y-F, Tang L, Qu X-J, Ying J-E, et al. The Chinese Society of Clinical Oncology (CSCO): Clinical guidelines for the diagnosis and treatment of gastric cancer, 2021. Cancer Commun (Lond). 2021;41(8):747–95. doi: 10.1002/cac2.12193 34197702 PMC8360643

[30] Dyshlovoy SA, Honecker F. Marine Compounds and Cancer: Updates 2022. Mar Drugs. 2022;20(12):759. doi: 10.3390/md20120759 36547906 PMC9783002

[31] Salih R, Bajou K, Shaker B, Elgamouz A. Antitumor effect of algae silver nanoparticles on human triple negative breast cancer cells. Biomed Pharmacother. 2023;168:115532. doi: 10.1016/j.biopha.2023.115532 37832405

[32] Al Monla R, Dassouki Z, Sari-Chmayssem N, Mawlawi H, Gali-Muhtasib H. Fucoidan and Alginate from the Brown Algae Colpomenia sinuosa and Their Combination with Vitamin C Trigger Apoptosis in Colon Cancer. Molecules. 2022;27(2):358. doi: 10.3390/molecules27020358 35056673 PMC8777791

[33] Shiau J-P, Chuang Y-T, Yang K-H, Chang F-R, Sheu J-H, Hou M-F, et al. Brown Algae-Derived Fucoidan Exerts Oxidative Stress-Dependent Antiproliferation on Oral Cancer Cells. Antioxidants (Basel). 2022;11(5):841. doi: 10.3390/antiox11050841 35624705 PMC9138104

[34] Hopkins AL. Network pharmacology: the next paradigm in drug discovery. Nat Chem Biol. 2008;4(11):682–90. doi: 10.1038/nchembio.118 18936753

[35] He N, Zhang J, Liu M, Yin L. Elucidating the mechanism of plasticizers inducing breast cancer through network toxicology and molecular docking analysis. Ecotoxicol Environ Saf. 2024;284:116866. doi: 10.1016/j.ecoenv.2024.116866 39178760

[36] Ooms LM, Binge LC, Davies EM, Rahman P, Conway JRW, Gurung R, et al. The Inositol Polyphosphate 5-Phosphatase PIPP Regulates AKT1-Dependent Breast Cancer Growth and Metastasis. Cancer Cell. 2015;28(2):155–69. doi: 10.1016/j.ccell.2015.07.003 26267533

[37] Sahlberg SH, Spiegelberg D, Glimelius B, Stenerlöw B, Nestor M. Evaluation of cancer stem cell markers CD133, CD44, CD24: association with AKT isoforms and radiation resistance in colon cancer cells. PLoS One. 2014;9(4):e94621. doi: 10.1371/journal.pone.0094621 24760019 PMC3997403

[38] Etemadmoghadam D, Bowtell D. AKT1 gene amplification as a biomarker of treatment response in ovarian cancer: mounting evidence of a therapeutic target. Gynecol Oncol. 2014;135(3):409–10. doi: 10.1016/j.ygyno.2014.11.007 25498304

[39] Zhang X, Wang S, Wang H, Cao J, Huang X, Chen Z, et al. Circular RNA circNRIP1 acts as a microRNA-149-5p sponge to promote gastric cancer progression via the AKT1/mTOR pathway. Mol Cancer. 2019;18(1):20. doi: 10.1186/s12943-018-0935-5 30717751 PMC6360801

[40] Hoogenboezem EN, Duvall CL. Harnessing albumin as a carrier for cancer therapies. Adv Drug Deliv Rev. 2018;130:73–89. doi: 10.1016/j.addr.2018.07.011 30012492 PMC6200408

[41] Chen L, Wei W, Sun J, Sun B, Deng R. Cordycepin enhances anti-tumor immunity in breast cancer by enhanceing ALB expression. Heliyon. 2024;10(9):e29903. doi: 10.1016/j.heliyon.2024.e29903 38720766 PMC11076851

[42] Nakazawa N, Sohda M, Yamaguchi A, Watanabe T, Saito H, Ubukata Y, et al. An Elevated Serum Lactate Dehydrogenase-to-albumin Ratio Is a Useful Poor Prognostic Predictor of Nivolumab in Patients With Gastric Cancer. Anticancer Res. 2021;41(8):3925–31. doi: 10.21873/anticanres.15188 34281855

[43] Kudou K, Saeki H, Nakashima Y, Kamori T, Kawazoe T, Haruta Y, et al. C-reactive protein/albumin ratio is a poor prognostic factor of esophagogastric junction and upper gastric cancer. J Gastroenterol Hepatol. 2019;34(2):355–63. doi: 10.1111/jgh.14442 30119141

[44] Zhou J, Ottewell PD. The role of IL-1B in breast cancer bone metastasis. J Bone Oncol. 2024;46:100608. doi: 10.1016/j.jbo.2024.100608 38800348 PMC11127524

[45] Huynh J, Chand A, Gough D, Ernst M. Therapeutically exploiting STAT3 activity in cancer - using tissue repair as a road map. Nat Rev Cancer. 2019;19(2):82–96. doi: 10.1038/s41568-018-0090-8 30578415

[46] Shen S, Niso-Santano M, Adjemian S, Takehara T, Malik SA, Minoux H, et al. Cytoplasmic STAT3 represses autophagy by inhibiting PKR activity. Mol Cell. 2012;48(5):667–80. doi: 10.1016/j.molcel.2012.09.013 23084476

[47] Gong J, Muñoz AR, Chan D, Ghosh R, Kumar AP. STAT3 down regulates LC3 to inhibit autophagy and pancreatic cancer cell growth. Oncotarget. 2014;5(9):2529–41. doi: 10.18632/oncotarget.1810 24796733 PMC4058024

[48] Li Z, Fan H, Chen W, Xiao J, Ma X, Ni P, et al. MicroRNA-653-5p Promotes Gastric Cancer Proliferation and Metastasis by Targeting the SOCS6-STAT3 Pathway. Front Mol Biosci. 2021;8:655580. doi: 10.3389/fmolb.2021.655580 33937336 PMC8082248

[49] Wang Z, Ni F, Yu F, Cui Z, Zhu X, Chen J. Prognostic significance of mRNA expression of CASPs in gastric cancer. Oncol Lett. 2019;18(5):4535–54. doi: 10.3892/ol.2019.10816 31611962 PMC6781674

[50] Petit AM, Rak J, Hung MC, Rockwell P, Goldstein N, Fendly B, et al. Neutralizing antibodies against epidermal growth factor and ErbB-2/neu receptor tyrosine kinases down-regulate vascular endothelial growth factor production by tumor cells in vitro and in vivo: angiogenic implications for signal transduction therapy of solid tumors. Am J Pathol. 1997;151(6):1523–30. 9403702 PMC1858348

[51] Shang L, Chen X, Zhu T, Chong S, Liu H, Huang W, et al. Cancer-Associated Fibroblast-Secreted Exosomes Promote Gastric Cancer Cell Migration and Invasion via the IL-32/ESR1 Axis. Appl Biochem Biotechnol. 2024;196(9):6045–58. doi: 10.1007/s12010-023-04782-6 38180644

[52] Lung DK, Reese RM, Alarid ET. Intrinsic and Extrinsic Factors Governing the Transcriptional Regulation of ESR1. Horm Cancer. 2020;11(3–4):129–47. doi: 10.1007/s12672-020-00388-0 32592004 PMC7384552

[53] Yang Z, An Y, Wang N, Dong X, Kang H. LINC02595 promotes tumor progression in colorectal cancer by inhibiting miR-203b-3p activity and facilitating BCL2L1 expression. J Cell Physiol. 2020;235(10):7449–64. doi: 10.1002/jcp.29650 32064615 PMC7496558

[54] Carneiro BA, El-Deiry WS. Targeting apoptosis in cancer therapy. Nat Rev Clin Oncol. 2020;17(7):395–417. doi: 10.1038/s41571-020-0341-y 32203277 PMC8211386

[55] Liu X, Jiang Q, Liu H, Luo S. Vitexin induces apoptosis through mitochondrial pathway and PI3K/Akt/mTOR signaling in human non-small cell lung cancer A549 cells. Biol Res. 2019;52(1):7. doi: 10.1186/s40659-019-0214-y 30797236 PMC6387544

[56] Hitosugi T, Kang S, Vander Heiden MG, Chung T-W, Elf S, Lythgoe K, et al. Tyrosine phosphorylation inhibits PKM2 to promote the Warburg effect and tumor growth. Sci Signal. 2009;2(97):ra73. doi: 10.1126/scisignal.2000431 19920251 PMC2812789

[57] Ohtsu A, Ajani JA, Bai Y-X, Bang Y-J, Chung H-C, Pan H-M, et al. Everolimus for previously treated advanced gastric cancer: results of the randomized, double-blind, phase III GRANITE-1 study. J Clin Oncol. 2013;31(31):3935–43. doi: 10.1200/JCO.2012.48.3552 24043745 PMC5950503

[58] Yu J, Song S, Jiao J, Liu X, Zhu H, Xiu L, et al. ZiYinHuaTan Recipe Inhibits Cell Proliferation and Promotes Apoptosis in Gastric Cancer by Suppressing PI3K/AKT Pathway. Biomed Res Int. 2020;2020:2018162. doi: 10.1155/2020/2018162 32382534 PMC7193275

[59] Liao Y, Chen W, Shi W, Zha H. Targeting cPLA2α inhibits gastric cancer and augments chemotherapy efficacy via suppressing Ras/MEK/ERK and Akt/β-catenin pathways. Cancer Chemother Pharmacol. 2021;88(4):689–97. doi: 10.1007/s00280-021-04322-1 34255137

[60] Ito K, Kitajima Y, Kai K, Matsufuji S, Yamada K, Egawa N, et al. Matrix metalloproteinase‑1 expression is regulated by HIF‑1‑dependent and epigenetic mechanisms and serves a tumor‑suppressive role in gastric cancer progression. Int J Oncol. 2021;59(6):102. doi: 10.3892/ijo.2021.5282 34738626 PMC8577796

[61] Sleep D. Albumin and its application in drug delivery. Expert Opin Drug Deliv. 2015;12(5):793–812. doi: 10.1517/17425247.2015.993313 25518870

[62] Jakkula E, Leppä V, Sulonen A-M, Varilo T, Kallio S, Kemppinen A, et al. Genome-wide association study in a high-risk isolate for multiple sclerosis reveals associated variants in STAT3 gene. Am J Hum Genet. 2010;86(2):285–91. doi: 10.1016/j.ajhg.2010.01.017 20159113 PMC2820168

[63] Hillmer EJ, Zhang H, Li HS, Watowich SS. STAT3 signaling in immunity. Cytokine Growth Factor Rev. 2016;31:1–15. doi: 10.1016/j.cytogfr.2016.05.001 27185365 PMC5050093

[64] Lacouture ME, Anadkat MJ, Bensadoun R-J, Bryce J, Chan A, Epstein JB, et al. Clinical practice guidelines for the prevention and treatment of EGFR inhibitor-associated dermatologic toxicities. Support Care Cancer. 2011;19(8):1079–95. doi: 10.1007/s00520-011-1197-6 21630130 PMC3128700

